# Tricin and tricin‐lignins in Medicago versus in monocots

**DOI:** 10.1111/nph.16827

**Published:** 2020-08-10

**Authors:** John Ralph

**Affiliations:** ^1^ Department of Biochemistry and DOE Great Lakes Bioenergy Research Center Wisconsin Energy Institute University of Wisconsin Madison WI 53726 USA

**Keywords:** alfalfa, flavonoids, hydroxylase enzymes, hydroxystilbenes, legume, lignification, monolignol biosynthetic pathway, monolignol conjugates

## Abstract

This article is a Commentary on Lui *et al*. (2020), **228**: 269–284.

The flavone tricin (Fig. 1) has become of significant interest recently for a number of reasons, not the least of which is that it is now found to be a monomer for the abundant lignin polymer in all grasses (Poaceae) in the commelinid monocots (del Río *et al*., 2012; Lan *et al*., 2015, 2016a,b). Tricin is scarcely found in dicots, but is known in alfalfa, *Medicago sativa* (Lan *et al*., 2016b). Tricin's rarity among the dicots, and the finding that it is also a lignin monomer in alfalfa, and the apparently different evolutionary course that allowed its biosynthesis, is the subject of a fascinating article in this issue of *New Phytologist*, Lui *et al*. (2020; pp. 269–284).

‘Medicago legumes have evolved their own distinct set of enzymes for tricin biosynthesis.’

**Fig. 1:**
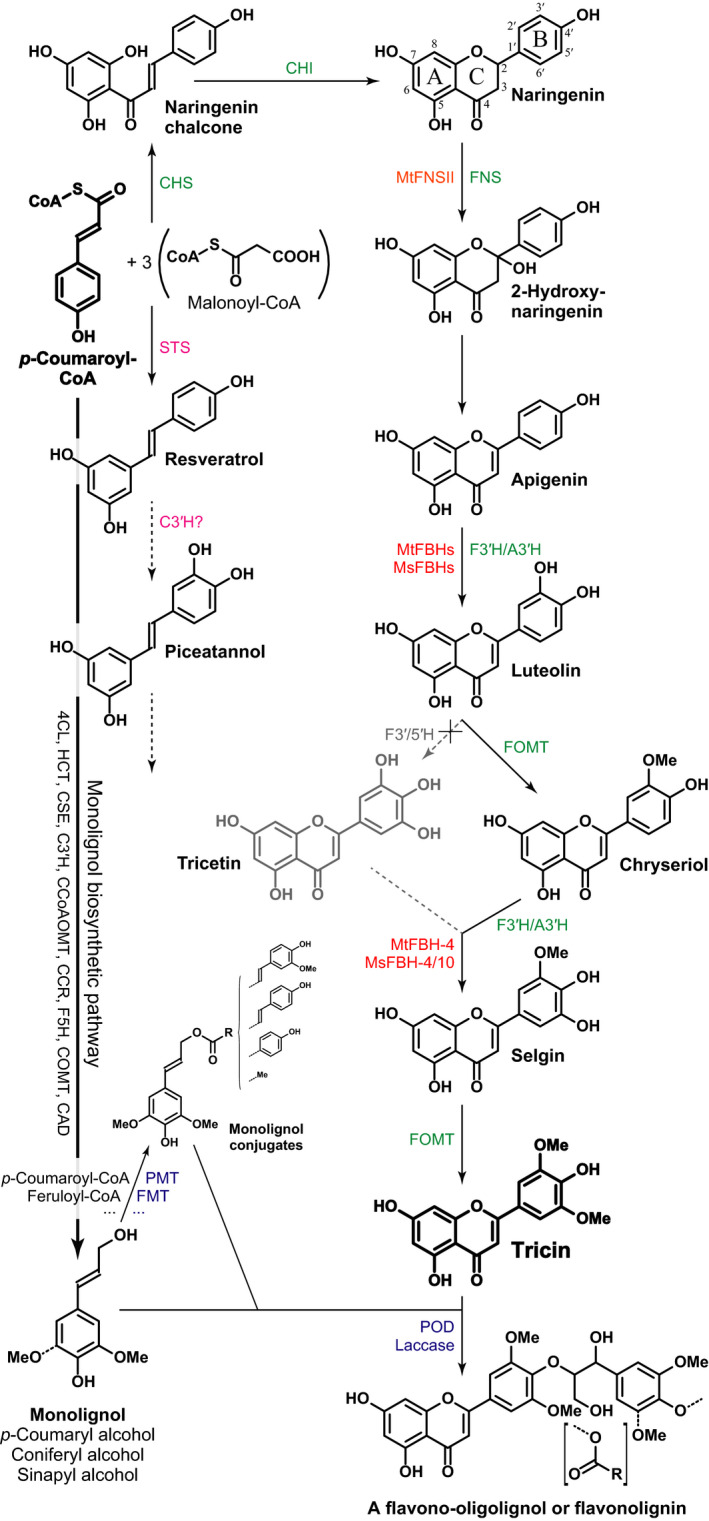
The tricin biosynthetic pathway from the key pathway‐branching intermediate, *p*‐coumaroyl‐CoA, with generic enzymes in grasses noted in green (Lam *et al*., 2015; Eloy *et al*., 2017), and in red are those from *Medicago sativa* (*Ms*) and *M. truncatula* (*Mt*) from the paper published in this issue of *New Phytologist* by Lui *et al*. (pp. 269–284). Note that the pathway via tricetin (light gray) has been ruled out in rice and presumably other grasses (Lam *et al*., 2015). As noted by Lui *et al*., the FNS enzyme (in orange) responsible for converting naringenin to apigenin (via 2‐hydroxynaringenin) was earlier determined to be via MtFNSII. Also shown is the general pathway into the hydroxystilbenes, including resveratrol and piceatannol, that have also served as lignin monomers in various endocarp tissue cell walls (del Río *et al*., 2017, 2020). It remains unclear whether piceatannol can derive from resveratrol, or if it is formed directly using STS from caffeoyl‐CoA, itself derived from *p*‐coumaroyl‐CoA via the monolignol pathway C3′H. On the left hand side (bold downward arrow) is the abbreviated pathway from *p*‐coumaroyl‐CoA to the monolignols (with enzymes simply shown, abbreviated, on the arrow); these pathways and enzymes have often been reviewed, most recently by Vanholme *et al*. (2019). The relatively newly understood pathways from the monolignols to their various acylated analogs are shown in the bottom left of the figure; BAHD transferases for *p*‐coumaroylation and feruloylation of monolignols have been revealed and reviewed (Vanholme *et al*., 2019); these acylated monolignols are also ‘monomers’ in lignification and in fact the available acetate and *p*‐coumarate conjugates in corn have been shown to combinatorially, along with the monolignols, couple with tricin to form variously acylated flavono‐oligolignols and polymeric flavonolignins (Lan *et al*., 2016a).

Lignin research has sporadically produced startling new insights, and at a rapid pace since the genetics revolution (Ralph *et al*., 2019; Vanholme *et al*., 2019). The ability to perturb the expression of single genes in the pathway gave researchers significant insight into the flexibility of lignification, the purely combinatorial chemical process of polymerizing monomers into the polymer, and necessitated a broad definitional change to include monomers beyond the canonical monolignols. Studies on various natural plants and different tissues have further continued to produce their own striking revelations. Perhaps none has been as remarkable as the discovery that tricin is a lignin monomer in a huge plant lineage, the commelinid monocots. Revealed in a paper from an ostensibly unassuming study characterizing wheat straw lignin, tricin (Fig. 1) was validated as a lignin component, and its integration into the polymer was indicated and then established beyond reasonable doubt (del Río *et al*., 2012). Although it could have been predicted from lignification's clear metabolic malleability (Ralph *et al*., 2019), tricin was the first phenolic component biosynthesized from outside the phenylpropanoid pathway to be established as an authentic lignin monomer, copolymerizing alongside the canonical monolignols, albeit joining a slew of other phenolics originating from the lignin pathway (Ralph *et al*., 2019; Vanholme *et al*., 2019). Tricin's role as a monomer has since been augmented by a set of hydroxystilbenes, also from a combination of the shikimate‐derived phenylpropanoid and acetate/malonate‐derived polyketide pathways (Fig. 1), and beyond (del Río *et al*., 2017, 2020).

These discoveries exploited the power of, and advances in, metabolite profiling and nuclear magnetic resonance (NMR) methods to elucidate the ‘new’ structures in the lignins, and their derivation, as well as to establish their true incorporation into the polymer (Lan *et al*., 2015, 2016a). In addition, the reevaluation of novel products in traditional and new analyses facilitated the discoveries and helped prove a monomer's incorporation into lignin. New products, many of which have become valuable markers, from analytical thioacidolysis first established the incorporation into lignins of hydroxycinnamaldehydes (from incomplete reduction to the monolignols), caffeyl alcohol and 5‐hydroxyconiferyl alcohol (from incomplete methylation), and hydroxycinnamates, as reviewed in Ralph *et al*. (2004). Development and deployment of new methods may be key to revealing new pathways. For example, it was not until after attempting to introduce novel monolignol ferulate conjugates (Fig. 1) into lignins, and the development of a method to establish success, was it revealed that such components were ‘always’ being incorporated into lignification in a range of native plant lines (Wilkerson *et al*., 2014; Karlen *et al*., 2016). Until that point, their role had gone unnoticed simply because no definitive assay existed for determining not just the existence of such conjugates but, importantly, that they were being integrally incorporated into the polymer during lignification.

Tricin's discovery in monocot lignins also added to the long‐appreciated realization that grasses, and commelinid monocots in general, are special in the plant kingdom. For evolutionary reasons about which we can only superficially speculate, grasses have at least four notable plant cell wall traits, none of which is unique but their combination is striking. (1) Commelinids have ferulates (and *p*‐coumarates) specifically attached to arabinoxylans (acylating the C5‐OH of arabinosyl units on (glucurono)arabinoxylans), that function to provide powerful mechanisms to strengthen the cell wall by cross‐linking both polysaccharides (with each other) and polysaccharides with lignin (Ralph, 2010). (2) Grasses have hydroxycinnamates acylating the lignins, now established to arise via the pre‐acylation of monolignols to produce conjugates that are then sent to the wall for otherwise normal lignification (Fig. 1, bottom left), as reviewed (Ralph, 2010). *p*‐Coumarates had long been known, and had been established to arise from monolignol *p*‐coumarate conjugates, but monolignol ferulates as authentic lignin precursors have been only more recently discovered (Karlen *et al*., 2016). (3) Not to leave out the polysaccharides, grasses contain mixed‐linkage (1→3,1→4) glucan polysaccharides (Henry & Harris, 1997). (4) As most recently revealed, grass lignins also contain tricin that resulted from its integration into the radical coupling process of lignification. Tricin levels can be as high as *c*. 3.3 wt% of the lignin (Lan *et al*., 2016b), striking levels given that tricin is capable only of starting a lignin chain and mechanistically cannot inculcate itself into an already growing polymer chain. With some 100 million dry tons (MT) of corn stover produced annually in the United States and an anticipated 250 MT augmented by miscanthus, switchgrass, and wheat straw under a given pricing scenario (according to the Department of Energy’s 2016 ‘*Billion‐Ton Report*’, https://www.energy.gov/eere/bioenergy/2016‐billion‐ton‐report), and at a nominal 15% lignin, this translates to some 38 MT of lignin that, at just 1.5 wt% tricin, contains over half a MT of tricin. Releasing that component from its polymer efficiently and economically is a serious challenge, but a currently low‐availability yet potentially valuable pharmaceutical, nutraceutical, and agricultural‐chemical product is suddenly recognized as being accessible in huge quantities.

It is with this background that we come back to the presence of tricin in dicots. As the paper in question notes, alfalfa *(Medicago sativa*) and its related *M. truncatula*, are rare dicots in possessing tricin in their lignins. The research groups involved have built upon substantial work on tricin in grasses, well covered in the paper by Lui *et al*., which has produced considerable insights into the pathways in grasses, and the genes/enzymes involved. The current paper presents a remarkable array of work aimed at elucidating the genes/enzymes involved in allowing Medicago to biosynthesize tricin and to use it in lignification. The paper details how the hydroxylase genes/enzymes appear to have evolved; these are crucial to hydroxylate and then methylate, that is, to methoxylate, the two carbons *ortho* to the B‐ring phenol (and often, as with the monolignols, referred to as being *meta* to the 3‐carbon aromatic sidechain) of the parent flavonoid naringenin to produce tricin as the ultimate product (Fig. 1). The authors have left essentially no question unaddressed or unanswered, and included approaches that might not have been required to produce an already compelling paper on a significant study, but add to the rigor and definitiveness of their findings. The paper itself should be consulted for the details because it is particularly well laid out and explained, and highlights are clearly made in the Summary section and in the carefully crafted headings in the main paper. In total, these findings reveal how Medicago has achieved the biosynthesis of tricin, including for its use in lignification, and how the process differs from, and is therefore evolutionarily independent of, that in the grasses. Incidentally, the authors are unconcerned about the ability of such phenolics to be ‘transported’ to the wall. As has been noted for a number of engineered products, or for those noncanonical monomers arising from truncation of the monolignol pathway and producing intermediates, or products therefrom, that enter into lignification, they ‘all’ make it to the wall even in newly created transgenics without the benefit of evolution. The logical conclusion is that transporters are either remarkably promiscuous or they are not needed at all. The case for passive transport of such compounds across membranes has recently been made (Vermaas *et al*., 2019). It only remains unfortunate that we cannot ask Medicago dicots, or the monocots for that matter, why they ‘thought’ it was advantageous to produce tricin!

The ‘new’ hydroxylase genes/enzymes revealed in Lui *et al*. will likely have value as researchers seek to introduce efficient pathways for introducing tricin into plant lines that currently do not possess it or use it in their lignification, for the prospect of either (or both) improving biomass conversion ease by lowering the recalcitrance attributable to lignin and toward valorizing lignin. To the extent that tricin, or other valuable flavonoid precursors along the pathway, are recoverable from the polymer, the realization that such valuable components may be associated with the currently underutilized and undervalued lignin, and on a scale never previously imagined, provides new opportunities to valorize the lignin component of biomass in crops and trees destined for biofuels and commodity chemicals production.

## References

[nph16827-bib-0001] del Río JC , Rencoret J , Gutiérrez A , Elder T , Kim H , Ralph J . 2020 Lignin monomers from beyond the canonical monolignol biosynthetic pathway: another brick in the wall. ACS Sustainable Chemistry & Engineering 8: 4997–5012.

[nph16827-bib-0002] del Río JC , Rencoret J , Gutiérrez A , Kim H , Ralph J . 2017 Hydroxystilbenes are monomers in palm fruit endocarp lignins. Plant Physiology 174: 2072–2082. 28588115 10.1104/pp.17.00362 PMC5543948

[nph16827-bib-0003] del Río JC , Rencoret J , Prinsen P , Martínez ÁT , Ralph J , Gutiérrez A . 2012 Structural characterization of wheat straw lignin as revealed by analytical pyrolysis, 2D‐NMR, and reductive cleavage methods. Journal of Agricultural and Food Chemistry 60: 5922–5935. 22607527 10.1021/jf301002n

[nph16827-bib-0004] Eloy NB , Voorend W , Lan W , de Lyra Soriano Saleme M , Cesarino I , Vanholme R , Smith RA , Goeminne G , Pallidis A , Morreel K , *et al* 2017 Silencing CHALCONE SYNTHASE in maize impedes the incorporation of tricin into lignin and increases lignin content. Plant Physiology 173: 998–1016. 27940492 10.1104/pp.16.01108 PMC5291018

[nph16827-bib-0005] Henry RJ , Harris PJ . 1997 Molecular distinction between monocotyledons and dicotyledons: more than a simple dichotomy. Plant Molecular Biology Reporter 15: 216–218.

[nph16827-bib-0006] Karlen SD , Zhang C , Peck ML , Smith RA , Padmakshan D , Helmich KE , Free HCA , Lee S , Smith BG , Lu F *et al* 2016 Monolignol ferulate conjugates are naturally incorporated into plant lignins. Science Advances 2: 1600391–1600399. 10.1126/sciadv.1600393 PMC5065250 27757415

[nph16827-bib-0007] Lam PY , Liu HJ , Lo C . 2015 Completion of tricin biosynthesis pathway in rice: cytochrome P450 75B4 is a unique chrysoeriol 5′‐hydroxylase. Plant Physiology 168: 1527–1536. 26082402 10.1104/pp.15.00566 PMC4528758

[nph16827-bib-0008] Lan W , Lu F , Regner M , Zhu Y , Rencoret J , Ralph SA , Zakai UI , Morreel K , Boerjan W , Ralph J . 2015 Tricin, a flavonoid monomer in monocot lignification. Plant Physiology 167: 1284–1295. 25667313 10.1104/pp.114.253757 PMC4378158

[nph16827-bib-0009] Lan W , Morreel K , Lu F , Rencoret J , del Rio JC , Voorend W , Vermerris W , Boerjan W , Ralph J . 2016a Maize tricin‐oligolignol metabolites and their implications for monocot lignification. Plant Physiology 171: 810–820. 27208246 10.1104/pp.16.02012 PMC4902589

[nph16827-bib-0010] Lan W , Rencoret J , Lu F , Karlen SD , Smith BG , Harris PJ , del Rio JC , Ralph J . 2016b Tricin‐lignins: occurrence and quantitation of tricin in relation to phylogeny. The Plant Journal 88: 1046–1057. 27553717 10.1111/tpj.13315

[nph16827-bib-0011] Lui ACW , Lam PY , Chan KH , Wang L , Tobimatsu Y , Lo C . 2020 Convergent recruitment of 5′‐hydroxylase activities by CYP75B flavonoid B‐ring hydroxylases for tricin biosynthesis in *Medicago* legumes. New Phytologist 228: 269–284. 10.1111/nph.16498 32083753

[nph16827-bib-0012] Ralph J . 2010 Hydroxycinnamates in lignification. Phytochemistry Reviews 9: 65–83.

[nph16827-bib-0013] Ralph J , Lapierre C , Boerjan W . 2019 Lignin structure and its engineering. Current Opinion in Biotechnology 56: 240–249. 30921563 10.1016/j.copbio.2019.02.019

[nph16827-bib-0014] Ralph J , Lundquist K , Brunow G , Lu F , Kim H , Schatz PF , Marita JM , Hatfield RD , Ralph SA , Christensen JH *et al* 2004 Lignins: natural polymers from oxidative coupling of 4‐hydroxyphenylpropanoids. Phytochemistry Reviews 3: 29–60.

[nph16827-bib-0015] Vanholme R , De Meester B , Ralph J , Boerjan W . 2019 Lignin biosynthesis and its integration into metabolism. Current Opinion in Biotechnology 56: 230–239. 30913460 10.1016/j.copbio.2019.02.018

[nph16827-bib-0016] Vermaas JV , Dixon RA , Chen F , Mansfield SD , Boerjan W , Ralph J , Crowley MF , Beckham GT . 2019 Passive membrane transport of lignin‐related compounds. Proceedings of the National Academies of Sciences, USA 116: 23117–23123. 10.1073/pnas.1904643116 PMC6859372 31659054

[nph16827-bib-0017] Wilkerson CG , Mansfield SD , Lu F , Withers S , Park J‐Y , Karlen SD , Gonzales‐Vigil E , Padmakshan D , Unda F , Rencoret J *et al* 2014 Monolignol ferulate transferase introduces chemically labile linkages into the lignin backbone. Science 344: 90–93. 24700858 10.1126/science.1250161

